# Inverse Association between Methylation of Human Papillomavirus Type 16 DNA and Risk of Cervical Intraepithelial Neoplasia Grades 2 or 3

**DOI:** 10.1371/journal.pone.0023897

**Published:** 2011-08-24

**Authors:** Long Fu Xi, Mingjun Jiang, Zhenping Shen, Ayaka Hulbert, Xiao-Hua Zhou, Ying-Ying Lin, Nancy B. Kiviat, Laura A. Koutsky

**Affiliations:** 1 Department of Pathology, School of Medicine, School of Public Health, University of Washington, Seattle, Washington, United States of America; 2 Department of Epidemiology, School of Public Health, University of Washington, Seattle, Washington, United States of America; 3 Department of Biostatistics, School of Public Health, University of Washington, Seattle, Washington, United States of America; Institut Pasteur, France

## Abstract

**Background:**

The clinical relevance of human papillomavirus type 16 (HPV16) DNA methylation has not been well documented, although its role in modulation of viral transcription is recognized.

**Methods:**

Study subjects were 211 women attending Planned Parenthood clinics in Western Washington for routine Papanicolaou screening who were HPV16 positive at the screening and/or subsequent colposcopy visit. Methylation of 11 CpG dinucleotides in the 3′ end of the long control region of the HPV16 genome was examined by sequencing the cloned polymerase chain reaction products. The association between risk of CIN2/3 and degree of CpG methylation was estimated using a logistic regression model.

**Results:**

CIN2/3 was histologically confirmed in 94 (44.5%) of 211 HPV16 positive women. The likelihood of being diagnosed as CIN2/3 increased significantly with decreasing numbers of methylated CpGs (meCpGs) in the 3′ end of the long control region (*P*_for trend_ = 0.003). After adjusting for HPV16 variants, number of HPV16-positive visits, current smoking status and lifetime number of male sex partners, the odds ratio for the association of CIN2/3 with ≥4 meCpGs was 0.31 (95% confidence interval, 0.12–0.79). The proportion of ≥4 meCpGs decreased appreciably as the severity of the cervical lesion increased (*P*_for trend_ = 0.001). The inverse association remained similar when CIN3 was used as the clinical endpoint. Although not statistically significant, the ≥4 meCpGs-related risk reduction was more substantial among current, as compared to noncurrent, smokers.

**Conclusion:**

Results suggest that degree of the viral genome methylation is related to the outcome of an HPV16 cervical infection.

## Introduction

Human papillomavirus type 16 (HPV16) is the most carcinogenic type of HPV [1], [2]. It is also the type commonly present in healthy women [3], [4], [5]. Most infections are transient with only a small fraction leading to the development of cervical cancer and its precursor lesion, cervical intraepithelial neoplasia grades 2 or 3 (CIN2/3). Given a recent recommendation of adding a test for oncogenic HPV types to programs for cervical cancer control [6], [7], it is worthwhile to understand factors which might discriminate between infections that are neoplastic and those that are self-limited. Studies *in vitro* have shown that methylation of the HPV16 genome results in a substantial repression of viral transcription and DNA replication [8], [9], [10], [11]. Because the expression of HPV16 oncogenes is essential to initiate transformation of infected epithelial cells, viral DNA methylation is likely to be involved in the outcome of infection.

DNA methylation refers to the addition of a methyl group by DNA methyltransferases to position 5 of the ring of cytosine (C), which occurs predominantly in the position immediately preceding a guanosine (G) in the DNA sequence. This change forms methylated CpG dinucleotides (meCpG), which is a major epigenetic mechanism for controlling gene expression in mammalian cells [12], [13], [14]. Unlike the wealth of evidence concerning the impact of aberrant promoter methylation of various cellular genes on the development of cervical cancer and its precancerous lesions [15], [16], reports of the potential clinical relevance of HPV16 DNA methylation are rare. Findings from a few small studies are inconsistent, with lower levels of CpG methylation among women with, compared to those without, cervical precursor lesions found in some studies [17], [18], [19], [20] but not others [21], [22]. It remains largely undetermined whether other methylation-related exposures, such as age and smoking [23], [24], [25], [26], [27], play a role in potential risks posed by HPV16 DNA methylation.

In this study, we describe the patterns of meCpGs in the 3′ end of the long control region of the HPV16 genome among women with and without a histological diagnosis of CIN2/3.

## Materials and Methods

### Study subject and design

Study subjects were a subset of women who participated in a study of new screening strategies for cervical cancer prevention. A detailed description of the original study design and population is presented elsewhere [28]. Briefly, the original study population was composed of women seen at 1 of 3 Planned Parenthood clinics in Western Washington for routine Papanicolaou (Pap) screening. Women were eligible if they were between 18 and 50 years of age, sexually active, not pregnant or immunocompromised, and had no history of hysterectomy or treatment for CIN. Between January 1997 and August 2002, a total of 5,103 women were enrolled. All participants underwent the standardized screening procedure, including a questionnaire, gynecologic examination, and collection of cervical samples for thin-layer cytology and HPV DNA testing. HPV DNA was detected by polymerase chain reaction (PCR)-based reverse-line blot. Women were referred for colposcopy and biopsy if they had a cytologic diagnosis of atypical squamous cells of undermined significance (ASC-US) or greater, or a positive test for an oncogenic HPV type(s). In addition, a random sample (10%) of women with normal screening results was also invited to undergo colposcopy and biopsy. At the colposcopy visit, cervical samples were collected again for thin-layer cytology and HPV DNA testing. Colposcopically-guided biopsies of the visible lesions were obtained, or if there was no visible lesion, a biopsy was taken at the 12-o'clock position on the cervix. Endocervical curettage was performed if the colposcopy was unsatisfactory, the screening cytology was high-grade squamous intraepithelial lesion (HSIL) but no lesion was visible during the colposcopy examination, or the lesions extended into the endocervical canal. A treatment with loop electrosurgical excision procedure was offered to women with histologically-confirmed CIN2/3.

A study participant was eligible for the present study, if she underwent colposcopy and biopsy and had HPV16 DNA detected at the screening and/or colposcopy visit. In total, 327 (6.4%) of 5,103 initially screened women were positive for HPV16. Of them, 104 were excluded including 78 who did not return for colposcopy, one with an unsatisfactory histologic diagnosis, and 25 with infsufficient samples for methylation testing. We additionally excluded 12 women because of failure to PCR-generate target fragments from their samples for methylation analyses. This left 211 women in the analysis, including 33 positive for HPV16 at the screening visit alone, 34 at the colposcopy visit alone, and 144 at both. HPV16 DNA methylation was assayed in cervical samples collected at the colposcopy visit for all except for those who had HPV16 detected at the screening visit alone. The present study utilized existing data and cervical samples collected in the screening study. A written informed consent regarding use of samples for future research was obtained from all participants. The institutional review board at the University of Washington approved the protocols for this study.

### Laboratory methods

DNA was extracted and purified from an aliquot of 200 µl of cervical swab sample in Specimen Transport Medium with QIAamp DNA Mini kit (Qiagen, Valentia, CA) and suspended in 100 µl AE buffer (10 mM Tris·Cl, 0.5 mM EDTA, *pH* 9.0). Bisulfite treatment of 50 to 150 ng sample DNA, which converts all unmethylated cytosines to uracils but leaves methylated cytosines intact, was completed using EpiTect® Bisulfite kit (Qiagen) according to the manufacturer's instructions. SiHa cellular DNAs with and without a treatment by CpG methyltransferase SssI (New England Biolabs, Ipswich, MA) were included in each run of the assay as the methylated and unmethylated controls, respectively, to monitor the completeness of bisulfite conversion.

The modified DNAs were PCR-amplified with a pair of primers (forward, 5′-tagttttatgttagtaattatggtt; reverse, 5′-tattaaaagagaattgtaatgttttaggat) for the 3′ end of the long control region of the HPV16 genome during which uracils were converted to thymines. We chose this region as a target because it is the main part of the viral regulatory region, which controls transcription of the E6 and E7 oncogenes. The reaction was setup in a total volume of 25 µl containing 1 µl sample DNA, 0.2 mM dNTPs, 0.5 µM primers, 2.5 mM MgCl2, 1× buffer II, and 1.25 unit AmpliTaq Gold DNA polymerase (Applied Biosystems, Foster City, CA). PCR amplification was carried out with the following profile: 94°C for 9 minutes to activate the polymerase; 44 cycles at 94°C for 30 seconds, 55°C for 30 seconds, and 68°C for 45 seconds; and a 7-minute terminal extension at 68°C.

The PCR-generated DNA fragments were cloned into plasmids using a TOPO TA Cloning kit according to the protocol recommended by the manufacturer (Invitrogen, Carlsbad, CA). Clones were screened by colony PCR followed by electrophoreses on the agarose gel to examine presence of target inserts. Plasmid DNAs from each clone were purified with a QIAprep Spin Miniprep Kit (Qiagen). The purified DNA templates were sequenced using a BigDye™ Sequencing kit (Applied Biosystems). DNA sequences were analyzed using the Sequencer™ package (Gene Codes Corp., Ann Arbor, MI), which shows cytosine if the original cytosine is methylated or thymine if the original cytosine is unmethylated. We selected 3 clones per sample for sequencing. A CpG site was considered methylated, if ≥1 clone displayed a methylated cytosine at this site.

### Statistical analysis

The main goal of the analyses is to examine the effects of HPV16 DNA methylation on the development of histologically confirmed cervical lesion. The 3′ end of the long control region covers 11 CpG sites: 5 (positions 7535, 7554, 7677, 7683, and 7695) within the enhancer region, 1 (position 7862) within the viral replication origin, and 5 (positions 31, 37, 43, 52, and 58) within the promoter region [18]. The number of meCpGs was categorized as 0, 1, 2–3 and ≥4 to reflect the extensiveness of methylation in the long control region and 0, 1, ≥2 to reflect the extensiveness of methylation in the enhancer or promoter region. One sample displayed a C-to-G alteration at position 43 in 2 clones and a methylated cytosine in the third. A meCpG at position 43 was assigned to this sample. Our HPV16 variant data suggest that most African variants have a C-to-T alteration at position 31. When this CpG site was excluded from the analysis for samples positive for the African variants (n = 13), the results remained similar (data not shown).

CIN2/3, otherwise specified, was the primary endpoint in all analyses. For women with more than 1 histologic diagnosis (due to either repeated biopsies or tissues from different sampling procedures), the most severe one was used as the final diagnosis. Two women had a diagnosis of CIN3 on their endocervical curettage sample and microinvasive or invasive squamous cell carcinoma on their loop electrosurgical excision sample. The methylation patterns in these two women were the same as those seen in CIN3 cases (data not shown). Thus, they were grouped with CIN3 cases for analyses.

A logistic regression model [29] was used to estimate odds ratios (ORs) and 95% confidence intervals (CIs) for the association between risk of CIN2/3 and degree of CpG methylation in various regions. The ORs were adjusted for HPV16 variants, number of HPV16 positive visits, current smoking status, and lifetime number of male sex partners. In this study, the methylation status was defined based on 3 clones per sample. To determine whether sequencing more clones per sample would alter estimates of risk association, we computed 95% CIs using a parametric bootstrap method with 10,000 repetitions of re-sampling 5 or 10 clones per specimen. Odds ratios were calculated 10,000 times; the lower and higher bounds of the 95% CI were given by the 250th and 9750th bootstrap odds ratios, respectively. In addition, we examined a potential of interaction between smoking and methylation using the likelihood ratio test comparing models with and without the interaction term.

A Cochran-Armitage test was used to assess the trend of decrease in proportions of CIN2/3 with increasing numbers of meCpGs in the 3′ end of the long control region and the trend of decrease in proportions of ≥4 meCpGs in this region with increasing severities of cervical lesion. We also assessed distributions of characteristics of women with versus without CIN2/3 by χ^2^ tests, including age at screening, race, lifetime number of male sex partners, having new male sex partners since screening, age at the first sexual intercourse, smoking status, current use of hormonal contraceptives, HPV16 variants, HPV16 positive visits, co-infection with other oncogenic types (i.e., HPV18/31/33/35/39/45/51/52/56/58/59/68), and cervical cytology. Characteristics were based on information at screening with the exception of new male sex partners and co-infection with other oncogenic types, which were based on information at the colposcopy visit and at the visit from which the sample was tested for methylation, respectively. A Student *t*-test was used to compare the mean lengths of intervals from the screening to colposcopy visit between women with and without CIN2/3. All statistical tests were conducted at the 5% two-sided significance level.

## Results

CIN2/3 was histologically confirmed in 94 (44.5%) of 211 HPV16-positive women. The mean length of intervals between the screening and colposcopy visits was 2.44 (SD, 3.24) months for women with CIN2/3 and 3.08 (SD, 3.94) months for those without (*p* = 0.20). As shown in Table 1, women with, compared to without, CIN2/3 were more likely to be positive for HPV16 non-European variants (*P* = 0.03), self-report as current smokers (*P* = 0.05), and have abnormal cytologic findings at screening (*P*<0.001) and a detectable HPV16 infection at both screening and colposcopy visits (*P* = 0.01).

**Table 1:** Characteristics of HPV16-positive women with and without CIN2/3.

Characteristic^a^	Without CIN2/3	With CIN2/3	
	no. (%)	no. (%)	*P*
Age at screening, years			0.56
18–20	33 (28.2)	19 (20.2)	
21–25	54 (46.2)	47 (50.0)	
26–30	20 (17.1)	17 (18.1)	
≥31	10 (8.5)	11 (11.7)	
Race^b^			0.65
Caucasian	84 (73.7)	64 (68.1)	
African-American	11 (9.6)	10 (10.6)	
Others	19 (16.7)	20 (21.3)	
Lifetime no. of male sex partners^c^			0.12
0–4	33 (28.2)	15 (16.1)	
5–9	39 (33.3)	36 (38.7)	
≥10	45 (38.5)	42 (45.2)	
Having new sex partners since screening			0.67
No	105 (89.7)	86 (91.5)	
Yes	12 (10.3)	8 (8.5)	
Age at the first sexual intercourse, years^d^			0.66
9–15<	47 (41.2)	39 (44.3)	
≥16	67 (58.8)	49 (55.7)	
Current hormonal contraceptive use			0.25
No	68 (58.1)	62 (66.0)	
Yes	49 (41.9)	32 (34.0)	
Current smoking			0.05
No	67 (57.3)	41 (43.6)	
Yes	50 (42.7)	53 (56.4)	
Coinfection with other oncogenic HPV types			0.61
No	81 (69.2)	62 (66.0)	
Yes	36 (30.8)	32 (34.0)	
HPV16 infections detected at			0.01
Screening visit alone	24 (20.5)	9 (9.6)	
Colposcopy visit alone	24 (20.5)	10 (10.6)	
Both	69 (59.0)	75 (79.8)	
HPV16 variant lineage^e^			0.03
European variants	103 (90.4)	75 (79.8)	
Non-European variants	11 (9.6)	19 (20.2)	
Cytologic findings at screening^f^			<0.001
Within normal limits	65 (56.5)	19 (20.2)	
ASC-US	23 (20.0)	15 (16.0)	
LSIL	16 (13.9)	14 (14.9)	
HSIL	11 (9.6)	46 (48.9)	

a Characteristics were based on information at the screening visit with the exceptions of having new sex partners since screening (collected at the colposcopy visit) and co-infection with other oncogenic HPV types, which was based on information at the visit from which the sample was assayed for methylation.

b Three women who did not provide information on race were excluded. The category of “Others” includes American Indian/Alaskan Native, Asian/Pacific Islander women and others.

c One woman who did not provide information on lifetime number of male sex partners was excluded.

d Nine women who did not provide information of age at the first sexual intercourse were excluded.

e Three women whose sample was inadequate for variant characterization were excluded.

f Two women whose sample was inadequate for cytologic evaluation were excluded.

### Patterns and frequencies of HPV16 DNA methylation

**Figure 1:**
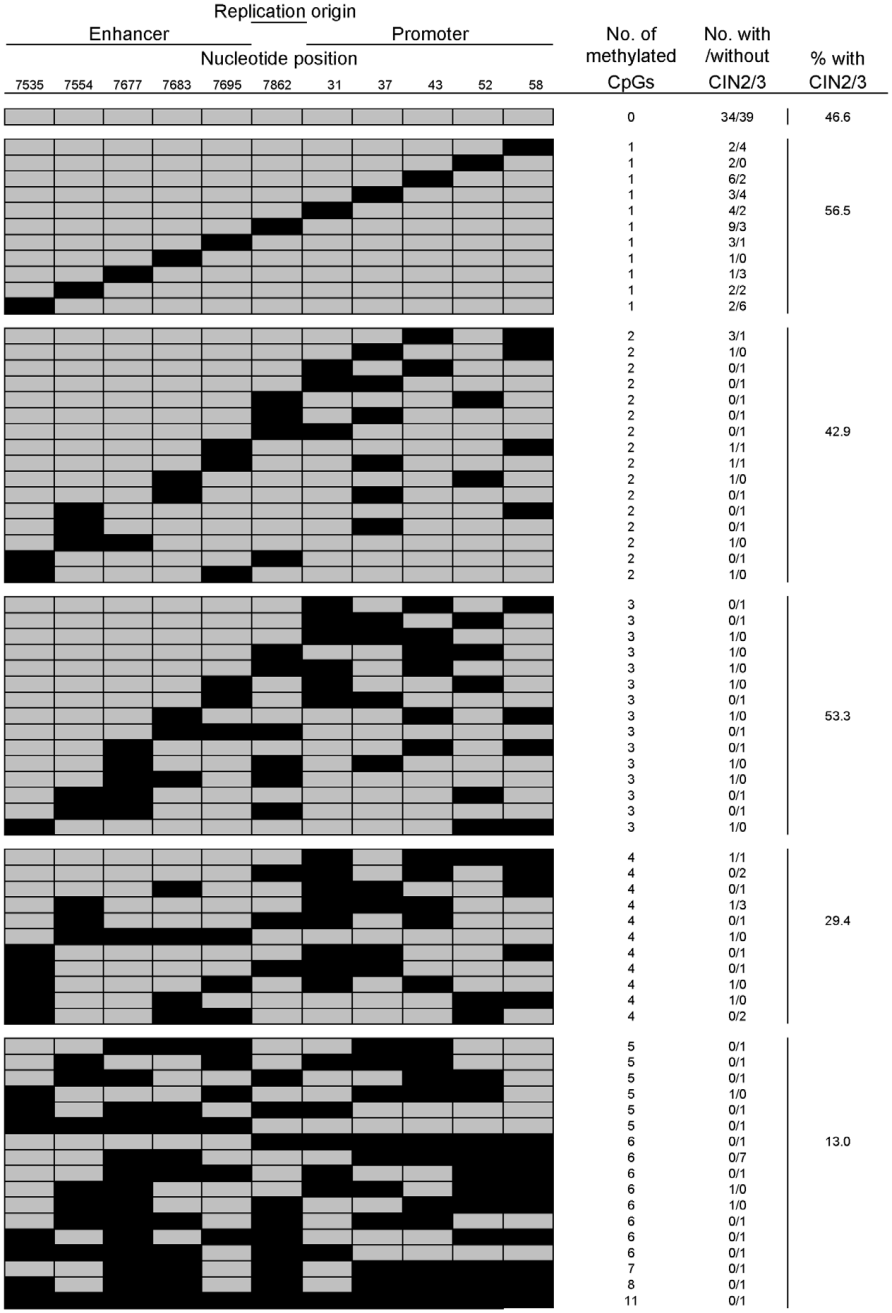
Patterns of CpG methylation in the 3′ end of the long control region of HPV16. Grey and black rectangles represent unmethylated and methylated CpGs, respectively. The numbers below the nucleotide position indicate a cytosine's position of each CpG dinucleotides.

Figure 1 summarizes the patterns of methylation in the 3′ end of the long control region. An assessment of a total of 2,321 CpGs (11 CpGs per sample for a total of 211 samples) revealed 357 (15.4%) meCpGs that were distributed in scattered patterns. The overall frequency of meCpGs in the region examined was significantly lower among women with, compared to without, CIN2/3 (114/1034 = 11.0% versus 243/1287 = 18.9%; *P*<0.001). Methylation of 1, 2, 3, 4 and ≥5 CpGs was detected in 62, 21, 15, 17, and 23 samples, respectively. The likelihood of being diagnosed as CIN2/3 increased significantly with decreasing numbers of meCpGs (*P*_for trend_ = 0.003).

**Figure 2:**
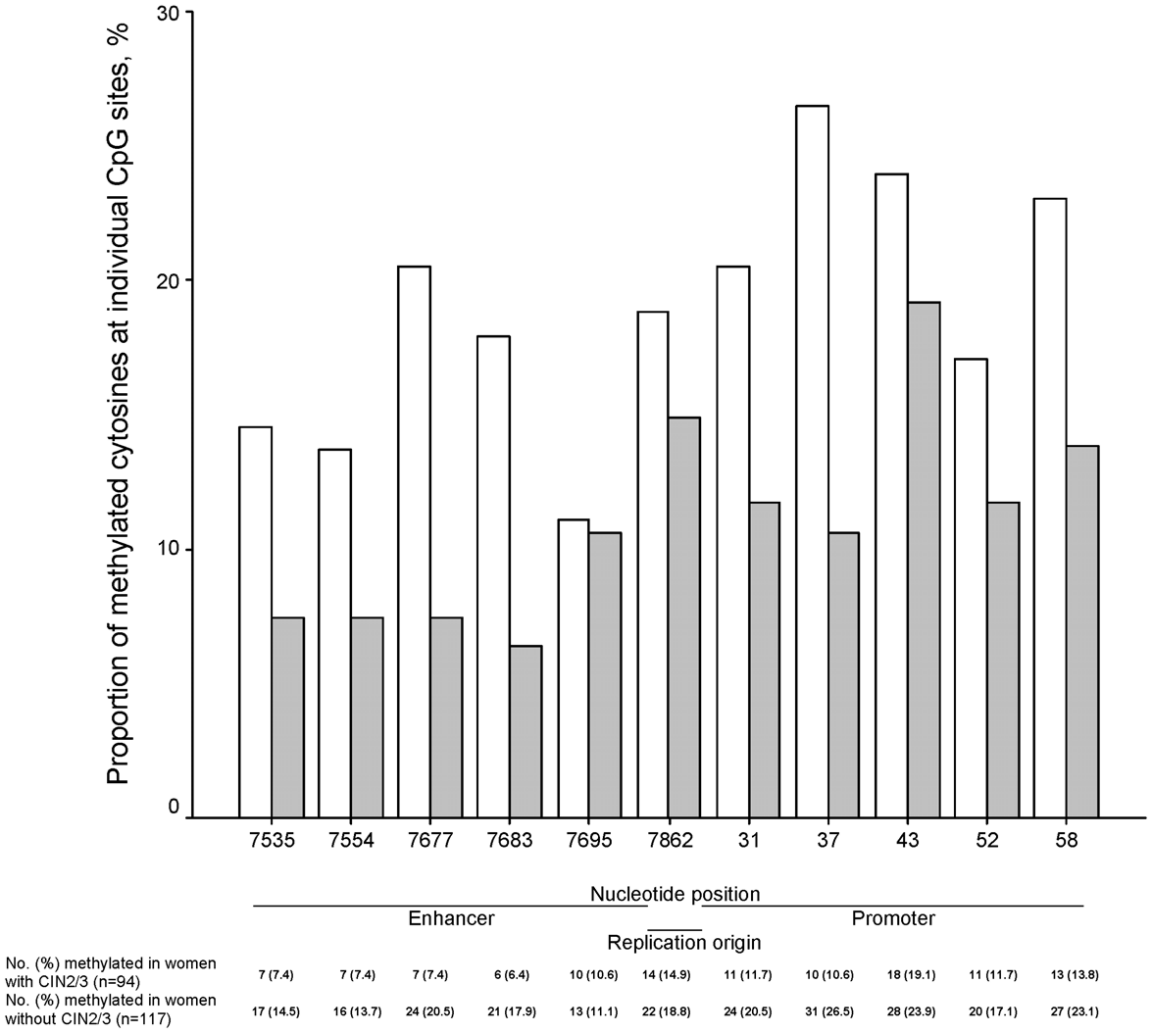
Proportions of the methylated cytosine at individual CpG dinucleotides between women with and without CIN2/3. Grey and white bars represent women with and without a diagnosis of CIN2/3, respectively. The numbers above the nucleotide position indicate a cytosine's position of each CpG dinucleotides in the 3′ end of the long control region of HPV16 genome.

The frequencies of methylation at individual CpG sites ranged from 6.4% at position 7883 to 19.1% at position 43 among women with CIN2/3 and from 11.1% at position 7695 to 26.5% at position 31 among those without CIN2/3 (Figure 2). It is visually apparent that women with CIN2/3 compared to those without had lower proportions of meCpGs across all sites except for position 7695. Despite an overall higher frequency of methylation in the promoter than in the enhancer (193/1055 = 18.3% versus 128/1055 = 12.1%, *p*<0.001), the pattern of lower frequencies of meCpGs among women with CIN2/3 compared to those without remained the same for both regions (37/470 = 7.9% versus 91/585 = 15.6%, *p*<0.001 for the enhancer; 63/470 = 13.4% versus 130/585 = 22.2%, *p*<0.001 for the promoter).

### Risk of CIN2/3 associated with degree of HPV16 DNA methylation

Table 2 shows the association of CIN2/3 with degree of HPV16 DNA methylation. The presence of ≥4 meCpGs in the 3′ end of the long control region was found in 8 (8.5%) of 94 women with CIN2/3 and 32 (27.4%) of 117 women without. After adjusting for HPV16 variants, number of HPV16 positive visits, current smoking status and lifetime number of male sex partners, the risk of CIN2/3 was significantly inversely associated with ≥4 meCpGs (OR _adjusted_ = 0.31; 95% CI, 0.12–0.79). Additional adjustment for age at screening or race did not alter the risk estimates appreciably (data not shown). The inverse association remained statistically significant or marginally statistically significant when the methylation status was based on CpGs in the enhancer and promoter, separately. As shown in Table 2, the 95% CIs estimated by a bootstrap with 10,000 repetitions of re-sampling 5 clones per sample were slightly narrow relative to the corresponding ones derived from the actual data. The patterns of the associations remained similar when 10 clones per sample were drawn for the bootstrap computation (data not shown).

**Table 2:** Odds ratios and 95% confidence intervals for the association of CIN2/3 with number of methylated CpGs in the regions analyzed.

No. of methylated	Without CIN2/3	With CIN2/3	Crude OR	Adjusted OR	95% CI by
CpGs within^a^	no. (%)	no. (%)	(95% CI)	(95% CI)^b^	bootstrap^c^
Long control region					
0	39 (33.3)	34 (36.2)	1.00	1.00	
1	27 (23.1)	35 (37.2)	1.49 (0.75–2.94)	1.49 (0.71–3.13)	0.98–1.89
2–3	19 (16.2)	17 (18.1)	1.03 (0.46–2.28)	0.89 (0.38–2.08)	0.63–1.23
≥4	32 (27.4)	8 (8.5)	0.29 (0.12–0.71)	0.31 (0.12–0.79)	0.14–0.35
Enhancer region					
0	66 (56.4)	68 (72.3)	1.00	1.00	
1	27 (23.1)	17 (18.1)	0.61 (0.31–1.22)	0.76 (0.36–1.59)	0.44–0.83
≥2	24 (20.5)	9 (9.6)	0.36 (0.16–0.84)	0.39 (0.16–0.94)	0.22–0.44
Replication origin					
0	95 (81.2)	80 (85.1)	1.0	1.0	
1	22 (18.8)	14 (14.9)	0.76 (0.36–1.57)	0.70 (0.32–1.52)	0.55–1.01
Promoter region					
0	58 (49.6)	56 (59.6)	1.00	1.00	
1	25 (21.4)	21 (22.3)	0.87 (0.44–1.73)	0.70 (0.33–1.48)	0.63–1.19
≥2	34 (29.1)	17 (18.1)	0.52 (0.26–1.03)	0.53 (0.26–1.11)	0.38–0.60

a Of 11 CpGs in the 3′ end of the long control region, 5 were within the enhancer region and 5 within the promoter region.

b Adjusted for HPV16 variants, number of HPV16 positive visits, current smoking status, and lifetime number of male sex partners.

c Upper and lower bounds of 95% CI estimated by bootstrapping 5 clones per sample for 10,000 times.

Thirty-three women (9 with CIN2/3 and 24 without) had HPV16 DNA detected at the screening visit alone. An exclusion of these women from the analysis did not appreciably alter the association of CIN2/3 with ≥4 meCpGs in the 3′ end of the long control region (OR _adjusted_ = 0.27; 95% CI, 0.09–0.82). Co-infection with other oncogenic HPV types was detected in 68 (32.2%) of 211 women. The association of CIN2/3 with ≥4 meCpGs remained similar when the analysis was restricted to women without co-infection of other oncogenic types (OR _adjusted_ = 0.15; 95% CI, 0.04–0.53). Given the significant association of ≥4 meCpGs in the 3′ end of the long control region with risk of CIN2/3, we further examined its impact on the lesion severity.

### HPV16 DNA methylation-related lesion severity

**Figure 3:**
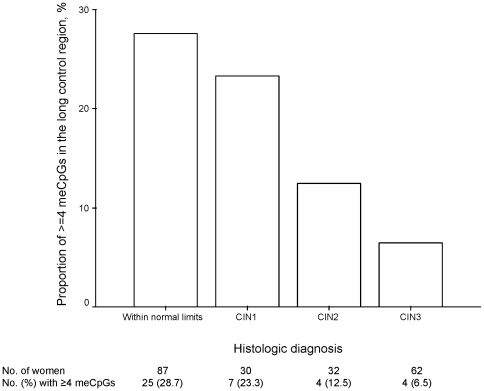
Proportions of ≥4meCpGs in the 3′ end of the long control region, stratified by cervical lesion severity.

Of 211 women who provided one or more tissue samples for diagnosis, CIN1, CIN2, or CIN3 was histologically confirmed in 30, 32, and 62 women, respectively. As shown in Figure 3, the proportions of ≥4 meCpGs in the 3′ end of the long control region decreased appreciably with an increase in lesion severity (*P*_for trend_ = 0.001). When CIN3 was used as the endpoint, the adjusted OR for the association with ≥4 meCpGs was 0.22 (95% CI, 0.07–0.74).

Data from the univariate analyses suggested a difference in CIN2/3 prevalence between current and noncurrent smokers. A question raised is whether the methylation-associated risk was modified by smoking habit.

### HPV16 DNA methylation-associated risk of CIN2/3, stratified by smoking status

The presence of ≥4 meCpGs in the 3′ end of the long control region was detected in 14 (13.6%) of 103 current smokers and 26 (24.1%) of 108 noncurrent smokers (*p* = 0.05). Although the decrease in ≥4 meCpGs-related risk was more substantial among current smokers (Table 3, OR = 0.12; 95% CI, 0.03–0.59) than in noncurrent smokers (OR = 0.40, 95% CI, 0.15–1.11), the test for interaction between smoking and methylation did not reach a statistically significant level (*P* = 0.21).

**Table 3:** Odds ratios and 95% confidence intervals for the association of CIN2/3 with ≥4meCpGs in the 3′ end of the long control region, stratified by current smoking status.

Current smoking	≥4meCpGs in the	Without CIN2/3	With CIN2/3	
	long control region	no. (%)	no. (%)	OR (95% CI)^a^
Yes	No	38 (42.7)	51 (57.3)	1.00
Yes	Yes	12 (85.7)	2 (14.3)	0.12 (0.03–0.59)
No	No	47 (57.3)	35 (42.7)	1.00
No	Yes	20 (76.9)	6 (23.1)	0.40 (0.15–1.11)

a The unadjusted OR was provided because of a small number of current smokers with ≥4meCpGs in the 3′ end of the long control region, particularly for those with a diagnosis of CIN2/3.

The risk association between CIN2/3 and ≥4 meCpGs in the 3′ end of the long control region did not differ meaningfully by age at screening, race, HPV16 variants, or number of HPV16 positive visits (data not shown).

## Discussion

In this study, we found that most CpGs in the 3′ end of the long control region were unmethylated; the likelihood of being diagnosed as CIN2/3 was significantly inversely associated with ≥4 meCpGs in this region. There was a trend showing an increase in lesion severity with a decrease in the proportion of ≥4 meCpGs. The inverse association was not explained by factors known to be relevant to the development of a cervical lesion including HPV16 variants, number of HPV16 positive visits, smoking status, and lifetime number of male sex partners nor by age and race. As shown by the analysis restricted to women without co-infection of other oncogenic HPV types, the association was also unlikely to result from effects of other HPV types. We are aware that methylation status might be unrelated to CIN2/3, if the infection became undetectable at the colposcopy visit. The association remained similar when the analysis was restricted to women who had HPV16 DNA detected at the colposcopy visit.

It should be pointed out that numbers of meCpGs defined in this study represented the methylation status of the infection rather than individual viral copies. While this analytic strategy minimizes ascertainment biases possibly introduced by counting exposures multiple times for a single infection, our measures were based on 3 clones per sample. Previous studies usually examined 5 clones per sample and treated each clone as a single data-point [18], [22], [30], [31]. Although an analysis of 10 clones per sample was reported, it included only 16 samples [32]. We acknowledge that because of a heterogeneous methylation status, more meCpGs would have been identified if more clones had been sequenced. However, this influence is likely to be non-differential because the present study assayed the same number of clones for virtually all samples. As suggested by the bootstrap analysis, sequencing more clones might improve a precision of the estimate by narrowing the confidence interval but not appreciably change the estimate of the risk association.

The present report is not the first to compare HPV16 DNA methylation status between women with and without a cervical lesion; but one of the first, if not the first study, to comprehensively address the impact of degree of CpG methylation on the development of CIN2/3 in a well defined screening population. In an earlier study of HPV16 methylation using methylation-specific restriction endonuclease digestion and direct sequencing of PCR products [17], CpG methylation in the long control region was found to be common in samples without abnormal cells, less common in samples with cells showing precancerous changes, and rare in samples with cancerous cells. In another study of HPV16 methylation (performed by subcloning and sequencing) [18], the frequency of methylation was found to be highest in specimens from cancer patients and lowest in women with cervical neoplasia. An association between lower levels of HPV16 methylation (hypomethylation) and cervical neoplasia has been reported by some studies [19], [20] but not others [21], [22]. The discrepancy of these results could be due to differences in study populations and approaches for methylation testing or a lack of sufficient statistical power.

The present study not only confirms the inverse association previously reported by some studies, but also extends previous findings by showing that the impact of methylation on risk of CIN2/3 depends on the number of detectable meCpGs in the 3′ end of the long control region. As compared to infections without detectable meCpG, risk of CIN2/3 was substantial for those with ≥4 meCpGs in the 3′ end of the long control region but not for those with a single meCpG. This methylation degree-related risk of CIN2/3 may in part explain discrepancies in previous reports because these studies usually dichotomized the status as methylated and unmethylated for risk assessment.

The underlying mechanism for the methylation-associated risk of CIN2/3 is presently unclear but it may result from a methylation-related alteration in the life-cycle of the virus. As shown in a schematic drawing by Ding et al [22], CpGs in the 3′ end of the long control region are at positions close to or within the binding sites for E2 proteins and various transcriptional factors. For example, positions 7535, 7554 and 7683 are close to AP1 and NF1 sites; position 7862 is close to CDP and AP1 sites and part of an E2 site; position 31 is within the SP1 site; and positions 37, 43, 52 and 58 are within the first two E2 binding sites next to the P97 promoter. Evidence from *in vitro* studies suggests that methylation of the HPV16 or HPV18 genome leads to a substantial repression of transcriptional activities by either directly or indirectly blocking the binding of the transcription factors [8], [33] or the E2 protein [9], [10] to elements in the long control region. Recent studies further showed a correlation between an increase in CpG methylation and decrease in expression of the E6 gene [20], [34]. Given the etiologic role of the oncogene's products, escape from this repression possibly by demethylation is likely to be a prerequisite for neoplastic progression.

The number of cases of cervical cancer in this study was too small (n = 2) to form a valid group for risk assessment. As noted, the highest frequency of HPV16 DNA methylation among cervical cancer patients was reported in most previous studies [18], [22], [35]. This does not conflict with our findings of hypomethylation-associated risk of CIN2/3. It is possible that the HPV genome in early stages of infection has to become demethylated in order to permit active viral transcription to initiate the transforming process. As carcinogenesis progresses, however, viral DNAs are frequently integrated into the host genome [36]. The heavy methylation seen among cancer patients might result from a host defense mechanism which senses the integrated viral genome as foreign [37], [38] and targets it for epigenetic modification. While the methylation resumes because of the integration, particularly for those tandem repeats, some HPV genomes remain unmethylated in cancer cells to maintain the transformed phenotype [39].

Consistent with findings reported by others [40], [41], [42], [43], [44], [45], [46], CIN2/3 occurred more frequently among current, as compared to noncurrent, smokers. Interestingly, the inverse association between risk of CIN2/3 and HPV16 DNA methylation appeared more substantial among women who self-reported as current smokers than among those who did not, although the test for interaction was not statistically significant. As shown in an *in vitro* study [47], exposure of cervical cells to Benzo[*a*]pyrene, a major carcinogen in cigarette smoke, stimulated higher levels of virion synthesis in HPV-infected cell lines. The smoke-associated increase in viral load was also observed in a population of HPV16-positive women [48]. Given an observation of lower proportion of infections with ≥4 meCpGs among current, as compared to noncurrent, smokers, we hypothesize that both cigarette smoke and HPV16 DNA methylation may play a role either independently or jointly in increasing CIN2/3 risk, perhaps by enhancing transcription and replication of the virus. The interplay of cigarette smoke and HPV16 DNA methylation on neoplastic progression warrants further investigation.

Several limitations of the study should be addressed. Despite the fact that this study included the largest number of infections to date, the number with ≥4 meCpGs among current smokers was small, particularly for those with a diagnosis of CIN2/3. Thus, the finding of a joint effect of smoking and methylation should be interpreted with caution. Secondly, cervical swab collects a mixed population of cells. Thus, the methylation detected in such a sample reflects the average of the HPV16 genomes from various cells. This as compared to use of microdissected uniform cells may attenuate a difference in methylation between women with and without CIN2/3, thereby leading to an underestimate of the risk association. Lastly, our study subjects were generally young. It is unclear whether the patterns of HPV16 methylation and their associations with risk of CIN2/3 would be the same for older women. However, due to a high proportion of young women attending Planned Parenthood Clinics both locally and nationally for Pap screening, our results are generalizable to a large segment of women between 18 and 50 years of age.

In summary, our data indicate that risk of CIN2/3 is inversely associated with degree of CpG methylation in the 3′ end of the long control region of the HPV16 genome. The association might be mediated through a methylation-related alteration in the biologic behavior of the virus.
